# Knowledge and Practices of Hand Washing among Health Professionals in Dubti Referral Hospital, Dubti, Afar, Northeast Ethiopia

**DOI:** 10.1155/2018/5290797

**Published:** 2018-11-22

**Authors:** Suoud Jemal

**Affiliations:** Department of Nursing, College of Medical and Health Sciences, Samara University, Afar, Ethiopia

## Abstract

**Introduction:**

Nosocomial infections due to poor hand hygiene are a major cause of increasing morbidity, mortality, and health care costs among hospitalized patients worldwide. Hand hygiene is mandatory to prevent the transmission of health care associated infections especially where infectious diseases are common like Sub-Saharan Africa. Hand washing compliance among health professionals in general is unacceptably low especially in developing countries like Ethiopia.

**Method:**

Institution-based cross-sectional study design was used to assess the knowledge and practice of hand washing among health professionals working in Dubti Referral Hospital. Structured self-administered pretested questionnaires were used. The data obtained were analyzed using SPSS version 23.

**Result:**

Out of 91 respondents, majority, 60 (65.9%), of them were knowledgeable and 31 (34.1%) were not knowledgeable. However, the majority of health professionals, 51 (56.0%), had poor practice and 40 (43.0%) of them had good practice of hand washing.

**Conclusion:**

Majority of health professionals were knowledgeable. However, they had poor practice of hand washing.

## 1. Introduction

Hand washing is the rubbing together of all surfaces and crevices of the hands using a soap or chemical and water. Hand washing should be performed after arriving at work, before leaving work, between client contacts, after removing gloves, when hands are visibly soiled, before eating, after excretion of body wastes (urination and defecation), after contact with body fluids, before and after performing invasive procedures, and after handling contaminated equipment. The exact duration of time required for hand washing depends on the circumstances. A washing time of 10 to 15 seconds is recommended to remove transient flora from the hands. High-risk areas, such as nurseries, usually require about a 2-minute hand wash. Soiled hands usually require more time [1].

Nosocomial infections due to poor hand hygiene are a major cause of increasing morbidity, mortality, and health care costs among hospitalized patients worldwide. The high prevalence of these infections, as high as 19%, in developing countries poses a challenge to health care providers [2]. Health care workers' hands are the most usual type of vehicle for transmission of health care associated infections. Pathogenic microorganisms can stay for 2-60 minutes on health care workers' hands [3]. Hand washing is the most simplest and effective measure to prevent infections. However, about 50% of health care associated infections occur due to hand of health care providers (HCPs) [4].

World Health Organization (WHO) introduced “My five moments for hand washing” to minimize problems related to hand washing. These five moments that call for the use of hand washing include the moment before touching a patient, before performing aseptic and clean procedures, after being at risk of exposure to body fluids, after touching a patient, and after touching patient surroundings [5].

As a study conducted to examine the hand hygiene knowledge, beliefs, and practices of Italian nursing and medical students with the aim of informing undergraduate curricula, a questionnaire was administered to convenience sample of 117 nursing and 119 medical students in a large university in Rome, Italy. The result of the study showed that nursing students' hand hygiene knowledge (F = 9·03(1,230); P = 0·003), percentage compliance (Z = 6·197; P < 0·001) and self-reported hand hygiene practices (F = 34·54(1,230); P < 0·001) were significantly higher than those of medical students. There were no statistically significant differences between hand hygiene beliefs [6].

A study done in General Hospital Ikot Ekpene, Akwa Ibom State, Nigeria, revealed that 82.4% of respondents had good knowledge of hand washing and 17.6% had poor knowledge. Observations on the practice of hand washing revealed that 42.2% of respondents always practiced hand washing and 34.3% practiced occasionally, while 23.5% never practiced hand washing [7].

In an observational study conducted among HPs in a Tertiary Hospital in Ghana, a hand washing compliance rate ranging from 9.2% to 57% among doctors and 9.6% to 54% among nurses was reported [8].

Hand washing compliance among health professionals in general is unacceptably low especially in developing countries like Ethiopia (range, 5%–89%; average, 38.7%) [9].

A study done in 2011 by Night project and Engender Health in Ethiopia showed that health care workers do not usually wash their hands on arrival to work place before putting on gloves [10].

A study conducted in Jimma University Hospital in Southwest Ethiopia also showed that hand washing practice by the nursing staff was inadequate. This study demonstrated that only 43.2% of the nursing staff practice adequate hand washing while 56.8% of them practice inadequate hand washing [11].

A study conducted in Health Institutions of Bahir Dar City Administration showed that 82.5% of health professionals had hand hygiene practice after completing the procedure they perform and about 50.8% wash their hand before the procedure. The overall hand hygiene practice score was 69.0% [12].

A study conducted in Shenen Gibe Hospital in Southwest Ethiopia showed that 68.8% had adequate practice and 82.97% were knowledgeable about hand washing [13].

## 2. Methodology

### 2.1. Study Area

The study was conducted in Dubti Referral Hospital. Dubti Referral Hospital is found in Dubti town, which is located in Northeastern Ethiopia, in Afar National Regional State, Zone 1, at a distance of 598km from Addis Ababa, and 10 km from Samara, the regional capital. Dubti Hospital is the referral hospital of Afar Region. Currently, there are a total of more than 400 health care workers in the hospital: composed of nurses, laboratory technicians, pharmacy technicians, physicians, midwives, etc.

### 2.2. Study Design

Institution-based descriptive cross-sectional study was used to assess the knowledge and practice of hand washing among health professionals in Dubti Referral Hospital.

### 2.3. Population

The source population for this study was all health professionals of Dubti Referral Hospital (including nurses, midwives, pharmacists, physicians, health officers, laboratory technicians, radiologists, and anesthetists).

### 2.4. Eligibility Criteria

#### 2.4.1. Inclusion Criteria

All health professionals of Dubti Referral Hospital who were available during data collection and interested in participating in the study were included.

#### 2.4.2. Exclusion Criteria

Health professionals who were not interested in participating in the study were excluded.

### 2.5. Sample Size Determination and Sampling Procedures

#### 2.5.1. Sample Size Determination

A single population proportion formula, [n = (Z *α*/2)^2^ p (1-p) / W^2^], was used to estimate the sample size. P=0.69 which was obtained from a study conducted in Bahir Dar city Administration in 2014, 95% confidence interval, and marginal error of 5% were used for sample size determination

The sample size was n_o_= (Z*α* /2)^2^(P) (q)/w^2^= (1.96)^2^(0.5) (0.5)/ (0.05)^2^=329. Since the study population was less than 10,000, correction formula was used

(1) nf=no1+no/Nnf=3291+329/109=82

The final sample size was 91 including 10% nonrespondent rate.

#### 2.5.2. Sampling Techniques and Procedure

Simple random sampling technique was used to select the study participants. The questionnaires were distributed to wards, emergency department, laboratories, outpatient departments, operation room, pediatrics, injection and dressing rooms, EPI unit, F.P unit, and others. Then they were filled by health professionals at their work places and collected by data collectors.

### 2.6. Data Collection Instrument

In conducting this study, structured self-administered questionnaires were used to collect the relevant data. The questionnaires contained closed and open ended questions about three different parts which included sociodemographic characteristics, knowledge of hand washing, and practice of hand washing among nurses. These questionnaires were distributed to wards, emergency department, laboratories, outpatient departments, operation room, pediatrics, injection and dressing rooms, EPI unit, F.P unit, and others. The distributed questionnaires were collected. In addition to this, observational checklist was used to collect data on practice of hand washing of health professionals.

### 2.7. Data Processing and Analysis

The collected data were checked for completeness and validity and analyzed by SPSS version 23. Finally, the result was presented by using frequency tables, graphs, and charts.

### 2.8. Operational Definition

**Knowledge** is defined as having adequate understanding about hand washing.

- **Knowledgeable**: earning score of 50% and above on the knowledge questions.
- **Not knowledgeable**: earning score less than 50% on the knowledge questions.

**Practice** is defined as an act of performing given procedure(s) according to a set standard.

- **Good practice**: study participants who responded to the practice questions ≥50% in line with the recommended hand washing practice were said to have good practice.
- **Poor practice**: study participants who responded to the practice questions < 50% in line with the recommended hand washing practice were said to have poor practice.

### 2.9. Data Quality Assurance

The collected data were checked regularly for clarity, completeness, consistency, accuracy, and validity. The prepared questionnaires were pretested on 5% of the total study population. The necessary correction was made on questionnaires that need correction accordingly and invalid questionnaires were removed before the actual data collection.

### 2.10. Ethical Consideration

Before the actual data collection, permission was asked and obtained from Dupti Referral Hospital administrators. The process of data collection was started after the health professionals were asked for willingness and verbal consent was obtained. Participants were also informed that participation was voluntary and that they could withdraw from the study at any stage if they desired without any penalty, and the information was kept confidential.

## 3. Results

### 3.1. Sociodemographic Characteristics of Study Participants

Out of 91 health professionals, 51 (56.0%) were males, 61 (67.0%) were single, 46 (50.6%) were Orthodox Christians, 45% were Muslims, and the rest (4.4%) were protestant. Majority (45.0%) were in the age of 21-25. Majority, 41 (45.0%), were nurses and 50 (55.0) held Diploma.  Table 1 shows the sociodemographic characteristics of the study participants.

**Figure 1:**
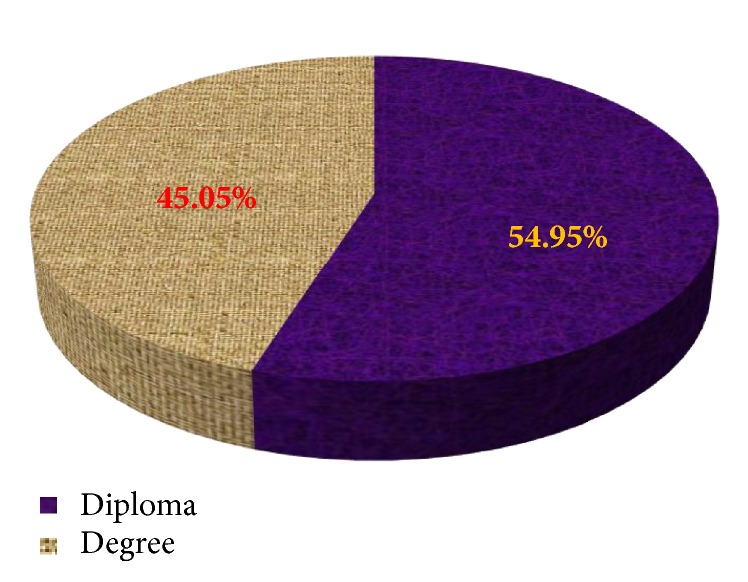
Frequency distribution of study participants by level of education, Dubti Referral Hospital, April 2016.

Figure 1 shows the frequency distribution of study participants by level of education in which 55.0% held diploma and the remaining 45.0% held degree.

**Figure 2:**
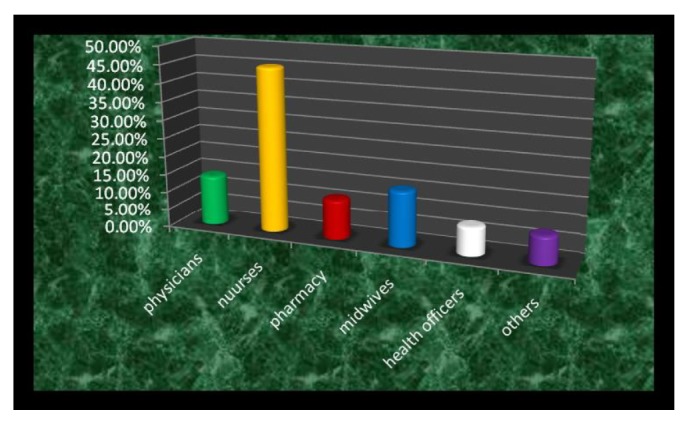
Frequency distribution of study participants by profession, Dubti Referral Hospital, April 2016.** Others**: laboratory technicians, radiologists, and anesthetists.

Figure 2 shows the frequency distribution of study participants by profession in which the majorities were nurses.

### 3.2. Knowledge of Study Participants towards Hand Washing

The knowledge of the respondents was assessed and categorized as knowledgeable and not knowledgeable. From 91 respondents, majority, 60 (66.0%), of them were knowledgeable and 31 (34.0%) of them were not knowledgeable about hand washing. 68 (74.7%) of the respondents did not know that hand washing was mandatory even if gloves were properly worn. Also, 46 (50.6%) of them did not know that hand washing was obligatory even for those cautious individuals.  Table 2 shows the knowledge score of the study participants.

**Figure 3:**
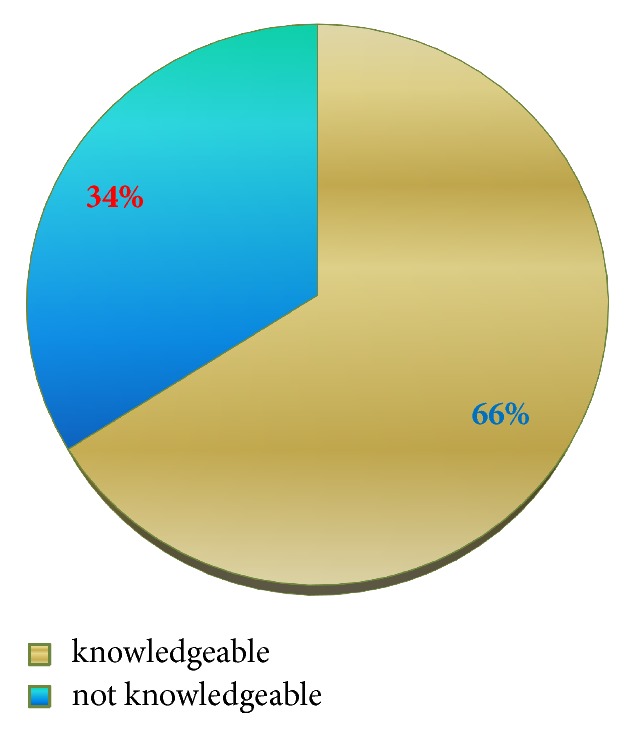
Distribution of the level of knowledge of hand washing, Dubti Referral Hospital, April 2016.

Figure 3 shows the level of knowledge in which majorities (66%) were knowledgeable.

### 3.3. Practices of Study Participants towards Hand Washing

From a total of 91 respondents, only 33 (36.3%) always washed their hands before clean and aseptic procedures. Only 18 (19.8%) of them always washed their hands before and after individual patient contact. 25 (27.5%) always used alcohol-based hand rub for hand hygiene. Also only 21 (23.1%) washed hands before contact with patients. However, 71 (78.0%) washed hands after contact with body secretions.  Table 3 shows practice of hand washing of study participants.

The practice of the respondents was assessed and categorized as good and poor practice. From 91 respondents, majority of them, 51 (56.0%), were categorized under poor practice and 40 (44.0%) of them were categorized under good practice of hand washing.

**Figure 4:**
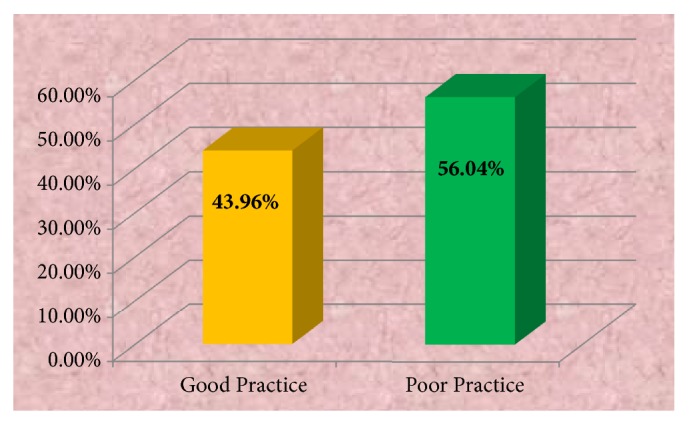
Distribution of hand washing of practice level in Dubti Referral Hospital among health professionals, April 2016.

Figure 4 shows the practice level of study participants in which 56.0% had good practice and the remaining 44.0% had poor practice.

*Reasons Given by Respondents for Not Practicing Hand Washing*. Out of 91 health care workers, 39 (42.9%) gave scarce of hand washing supplies, 23 (25.8%) gave work overload, and 29 (31.9%) gave shortage of time as a reason for not washing their hand. Out of 39 respondents, 26 (28.6%) complained of shortage of water; 8(8.8%) complained of shortage of soap; and 5 (5.5%) complained of shortage of antiseptic agents as scarce of hand washing supplies.

*Antiseptic Use in the Clinical Practice*. Out of the 91 respondents, 66 (72.5%) used soap and 25 (27.5%) used alcohol.

## 4. Discussion

In this study, out of 91 health professionals, 60 (65.93%) were knowledgeable and 31 (34.07%) were not knowledgeable. This result was lower than the result of a study done at Shenen Gibe Hospital, Southeast Ethiopia, in which 82.9% had good knowledge and 17.1% had poor knowledge [13], and comparable with the study done at Jimma University Hospital on hand washing among nursing staffs in which 71.6% had good knowledge while the rest 28.4% had poor knowledge [11]. This might be due to the time gap, homogeneity of the study participants, and status of the hospital.

Out of 91 respondents, 33 (36.26%) always wash their hands before performing aseptic and clean producers. This finding was lower than that in the study conducted at Bahir Dar City Administration Health Institutions, which was about 50.8%. But the overall of hand washing practice of this study finding was relatively similar to a study conducted in Bahir Dar City Administration [12], Shenen Gibe Hospital [13], and Southwest Nigeria Tertiary Hospital [14], which was 69%, 68.8%, and 69.9%, respectively. However, it was higher than a study conducted in Sri Lanka which showed that only 10% had overall good practices, while 27% had moderate practices and majority (62.5%) were seen to have poor hand hygiene practices [15].

This study showed that, out of the 91 respondents, 66 (72.53%) used soap and 25 (27.47%) used alcohol. This result was lower than the study conducted at Bahir Dar City Administration on hand washing [12] which showed that 98% used soap and alcohol and 2% used Savlon and more than one antiseptic agent. This might be due to difference in availability of antiseptic agents.

## 5. Conclusion

Based on this study, majority of the study participants were knowledgeable. However, they had poor practice of hand washing.

## 6. Recommendation

Dubti Referral Hospital should give attention to improving the knowledge and practice of those HCWs towards hand washing. Regular practice of hand washing requires supplies like soap, water, dry and clean towel, etc. depending on the type of procedure to be performed at all times. Therefore, the hospital and other concerned bodies should fulfill those necessary facilities to improve practice of hand washing. The hospital authorities are responsible for posting the general guidelines for the staff at each hand washing site. Finally the investigator of this study recommends further investigation to be done on the issue

## Figures and Tables

**Table 1:** Sociodemographic characteristics of health professionals in Dubti Referral Hospital, April 2016.

**S.N**	**Variable**		**Frequency**	**Relative frequency (**%**)**
**1**	Age	<21	8	8.8
		21-25	41	45.0
		26-30	29	31.9
		31-35	5	5.5
		>35	8	8.8

**2**	Sex	Male	51	56.0
		Female	40	44.0

**3**	Marital status	Single	61	67.0
		Married	28	30.8
		Divorced	2	2.2

**4**	Religion	Muslim	41	45.0
		Orthodox	46	50.6
		Protestant	4	4.4

**Table 2:** Knowledge of hand washing of study participants in Dubti Referral Hospital, April 2016.

**Variables **	**Response**					
	**Yes**		**No**		**I don't know**	
	**frequency**	**Relative frequency**	**frequency**	**Relative frequency**	**frequency**	**Relative frequency**
**Direct or indirect contacts are the most important routes for transmission of hospital-acquired infections**	76	83.5	11	12.0	4	4.4

**Proper and consistent hand washing prevents infections in health facilities **	77	84.6	12	13.2	2	2.2

**There is no need for hand washing for those who perform their activity with caution**	46	50.6	42	46.2	3	3.3

**There is no need of hand washing if gloves are properly worn**	68	74.7	17	18.9	6	6.6

**Health professionals should always wash their hands immediately when they arrive at health institutions**	52	57.1	36	39.6	3	3.3

**Hand hygiene should be practiced routinely even when gloves are worn**	48	52.8	33	36.3	10	11.0

**Effective hand washing consists of wetting, soaping, applying friction, rinsing and drying adequately**	71	78.0	13	14.3	7	7.7

**Hands should be washed at least for 10-15 seconds**	58	63.7	22	24.2	11	12.1

**Using disinfectants during hand washing decreases bacterial load on hands**	75	82.4	11	12.1	5	5.6

**Health professionals should wash their hands or use antiseptic hand rub before putting on or after removal of gloves**	73	80.2	18	19.8		

**Alcohol has the ability to eradicate micro-organisms compared to water**	69	75.8	17	18.7	5	5.6

**Hand washing is the single most effective mechanism to prevent spread of infection**	59	64.8	21	23.1	11	12.1

**Table 3:** Practice of hand washing of study participants in Dubti Referral Hospital, April 2016.

**Variable **	**Response**	**Frequency**	**Relative frequency (**%**)**
**Wash hands before contact with patients**	Always	21	23.1
	Usually	20	22.0
	Often	18	19.8
	Sometimes	27	29.7
	Never	5	5.5

**Wash hands after contact with patients**	Always	31	34.1
	Usually	20	22.0
	Often	8	8.8
	Sometimes	24	26.4
	Never	8	8.8

**Wash hands before and after contact with patients**	Always	18	19.8
	Usually	18	19.8
	Often	27	29.7
	Sometimes	13	14.3
	Never	15	16.5

**Wash hands after contact with body secretions**	Always	71	78.0
	Usually	11	12.1
	Often	9	9.9

**Wash hands before performing any clean and aseptic procedures**	Always	33	36.3
	Usually	33	36.3
	Often	9	9.9
	Sometimes	11	12.1
	Never	5	5.5

**Apply soap during hand washing**	Always	47	51.6
	Usually	19	20.9
	Often	13	14.3
	Sometimes	8	8.8
	Never	4	4.4

**Moisten hands under running water before applying soap**	Always	69	75.8
	Usually	13	14.3
	Often	9	9.9

**Use alcohol-based hand rub for hand hygiene**	Always	25	27.5
	Usually	17	18.7
	Often	16	17.6
	Sometimes	20	22.0
	Never	13	14.3

**Dry hands after hand washing**	Always	50	55.0
	Usually	19	20.9
	Often	11	12.1
	Sometimes	8	8.8
	Never	3	3.3

**Wash hands before leaving the hospital**	Always	70	76.9
	Usually	11	12.1
	Often	10	11.0

## Data Availability

The data used to support the findings of this study are available from the corresponding author upon request.
